# Radical Laparoscopic Pericystectomy of a Giant Hepatic Hydatid Cyst

**DOI:** 10.7759/cureus.71674

**Published:** 2024-10-17

**Authors:** Camila Sotomayor, Natalia Reyes, Eduardo Briceño, Martín Dib, Eduardo Viñuela, Jorge Martínez, Nicolás Jarufe

**Affiliations:** 1 Department of Hepatobiliary Surgery, Pontificia Universidad Católica de Chile, Santiago, CHL

**Keywords:** hepatic hydatidosis, laparoscopic technique, minimally invasive liver resection, pericystectomy, zoonosis

## Abstract

Hydatidosis is an endemic zoonotic disease with an uneven geographical distribution due to the varying abundance of its intermediate hosts, primarily cattle, and sheep in different regions, leading to a higher concentration of cases in livestock areas. Despite advancements in medical treatment and interventional radiology, surgery remains the treatment of choice for patients with hepatic hydatid cysts (HHC). Over the past decade, laparoscopic management of HHC has gained popularity; however, controversies persist regarding optimal patient selection, surgical techniques, and follow-up protocols. Conservative techniques, including capitonnage, partial cystectomy, puncture, and aspiration, have been described, while radical approaches such as pericystectomy and liver resection demonstrate superior management of the residual cavity and reduced recurrence rates. We present the case of a 52-year-old male presenting with right hypochondriac abdominal pain, whose CT scan revealed a giant, uncomplicated hepatic hydatid cyst in the right lobe. The video demonstrates a radical approach using subtotal pericystectomy for a giant hepatic hydatid cyst (>10 cm) located in the right hepatic lobe. The patient had an uneventful recovery and was discharged without complications on the third postoperative day.

## Introduction

Hydatid liver disease, also known as *echinococcosis*, is a zoonotic infectious disease caused by the larvae of tapeworms of the genus *Echinococcus* [1]. Two species primarily affect humans: *E. granulosus*, which causes cystic hydatidosis, and *E. multilocularis*, responsible for alveolar hydatidosis [2].

The liver is the most commonly affected organ, accounting for 50-70% of cases, followed by the lungs and spleen. Notably, cysts are more likely to involve the right lobe than the left, attributed to the pattern of portal blood flow [3].

The life cycle of the parasite involves two hosts: the definitive (or primary) host and the intermediate host, in which the disease develops. The adult form of the parasite resides in the intestines of definitive hosts, such as cats, dogs, and wolves, causing intestinal parasitosis without affecting other organs. These hosts excrete millions of eggs through their feces. Herbivorous animals, such as sheep, become intermediate hosts by ingesting contaminated vegetation. Humans may also become intermediate hosts through the consumption of food contaminated with these eggs. There is no human-to-human transmission [3].

In Chile, hydatidosis is an endemic zoonosis, with a national prevalence of five cases per 100,000 people and a higher prevalence of 48 cases per 100,000 in the Araucanía Region, located in southern Chile [4]. The higher prevalence of the disease in this region is likely attributable to the concentration of livestock farming. Cystic *echinococcosis* is usually asymptomatic or nonspecific. This is because the disease commonly remains unnoticed for several years due to the slow growth of cysts, which range from 1-2 mm to 10 mm per year [3]. As a result, the diagnosis is often made incidentally.

Symptoms arise from cyst expansion or the host's inflammatory response, leading to irritation of the adjacent parietal peritoneum. [1]. Furthermore, depending on the size and location, the cysts can compress adjacent structures, leading to abdominal pain [3].

Due to diverse mechanisms, complications arise in 20-40% of cases, encompassing bile duct damage (up to 42%); compression of the hepatic, portal, or vena cava, cyst rupture (1-8%); bacterial superinfection (7%); severe anaphylactic reactions (1%); and cystobronchial fistulas. Although these are rare, they can be fatal without early and appropriate treatment [5,6].

The disease diagnosis primarily relies on imaging outcomes, while epidemiological data, clinical observations, and serological tests also aid in confirming the diagnosis [7].

Routine laboratory workup may reveal eosinophilia and abnormal liver function tests; however, these findings are not specific to hydatid liver disease [2].

Imaging techniques are frequently employed to visualize the hydatid cyst and its contents. When used in combination, these methods are highly accurate and often enable a conclusive diagnosis. In addition, they can evaluate any complications associated with the cyst [2].

Ultrasound (US) is the first-line examination. ­It has a sensitivity of 90-95% and may allow the classification as active, transitional, or inactive. Computed tomography (CT) with intravenous contrast has higher sensitivity than US, reaching up to 95%. It helps to determine the size, number, and location of the cysts. It also shows extra-hepatic cysts better than US.

Currently, the Gharbi classification, which delineates five ultrasound stages, is the most commonly utilized. Another classification, established by the WHO-Informal Working Group on Echinococcosis (WHO/IWGE), differs from Gharbi's by categorizing cysts into initial undifferentiated cystic lesions and evolved cysts [2,8].

There are numerous debates in the literature regarding the most appropriate approach for managing liver hydatid disease. According to certain studies, open surgery has been advocated over medical therapy for the treatment of *echinococcosis*. However, recent WHO guidelines suggest treating uncomplicated liver hydatid cysts using the PAIR (puncture-aspiration-injection-reaspiration) procedure [9].

The conventional treatment globally recognized for this condition involves surgically removing the cyst entirely through open surgery, an option available for patients capable of tolerating the procedure. Advances in this area have progressively expanded, resulting in minimally invasive techniques like laparoscopy being utilized for cyst removal [10].

Surgical procedures, whether open surgery or laparoscopy, are divided into conservative and radical procedures. In the former, the preservation of liver parenchyma is essential, while the latter includes any type of resection such as pericystectomy [11], segmentectomy, and lobectomy [9,12].

In the last decade, laparoscopic treatment of hepatic hydatidosis has become more popular. The laparoscopic approach has been repeatedly shown to successfully remove liver hydatid cysts; however, the controversies surrounding the role of laparoscopy in the treatment of hepatic hydatid cysts remain unresolved, mainly due to limited global experience, diversity in surgical techniques employed, lack of uniformity in patient selection, and absence of an adequate follow-up protocol [12].

This case report was previously presented as a meeting video at the 2024 IHPBA World Congress, held in Cape Town, South Africa on May 15-18, 2024.

## Case presentation

We present the case of a 52-year-old male with no significant medical or surgical history, residing in a city in the northern region of the country, who presented with right hypochondriac abdominal pain. The pain had a gradual onset, was mild to moderate in intensity, and persisted for several hours without radiation. Vital signs were within normal limits. Abdominal examination revealed hepatomegaly approximately 3 cm below the right costal margin, which was firm in consistency and tender on palpation.

Significant laboratory findings included a white blood cell count of 11 x10^3^ uL with 74% neutrophils. Table 1 presents the laboratory results.

**Table 1:** Laboratory results

Tests	Result	Reference range
Hemoglobin	13.0 g/dl	12.5-16 g/dl
White cell count	11.0 x10^3^ uL	4.5-11 x10^3^ uL
Neutrophils	74%	50-70%
Lymphocytes	17.6%	25-40%
Monocytes	7%	4-12%
Eosinophils	0.6%	2-4%
Platelet count	356 x10^3^ uL	140-400 x10^3^ uL
Total bilirubin	0.4 mg/dL	0.2-1.0 mg/dL
Aspartate aminotransferase (AST)	61 UL	5-35 U/L
Alanine aminotransferase (ALT)	43 UL	5-35 U/L
Gamma-glutamyl transferase (GGT)	59 UL	5-40 UL
Alkaline phosphatase	197 UL	30-100 UL
Serum creatinine	0.65 mg/dL	0.5-0.9 mg/dL
International normalized ratio (INR)	1.0	

**Figure 1:**
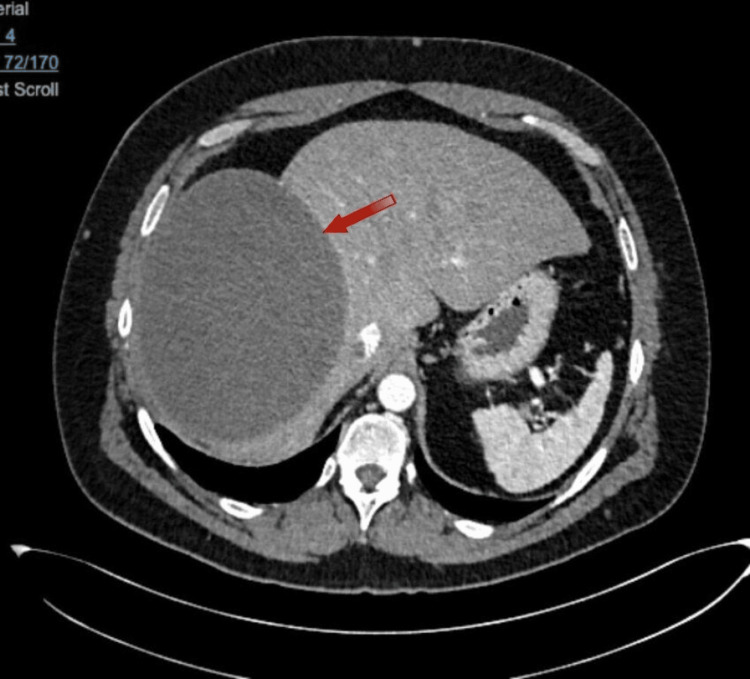
CT axial plane There is a presence of a giant, uncomplicated cyst in the right lobe.

**Figure 2:**
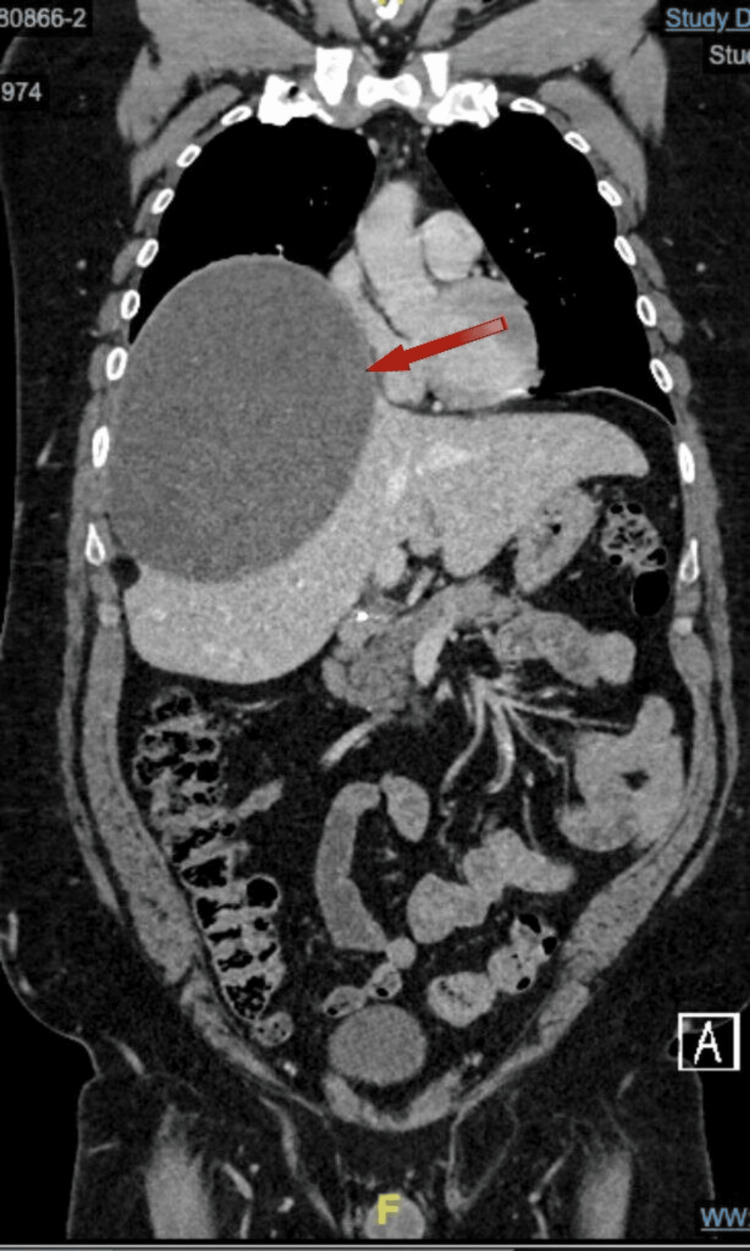
CT coronal plane

CT (Figure 1, Figure 2) indicated the presence of a giant, uncomplicated cyst in the right lobe, Approximately 16.6 x 13.4 x 17.5 cm in its longitudinal, anteroposterior, and transverse axes, respectively, indicating elevation of the right hemidiaphragm. No solid nodules, thick septa, or calcifications were observed in its thickness. The differential diagnosis included (among other less common possibilities) a hemorrhagic cystic lesion and a hydatid cyst, so it was suggested to complement the study with an abdominal MRI for better characterization. 

**Figure 3:**
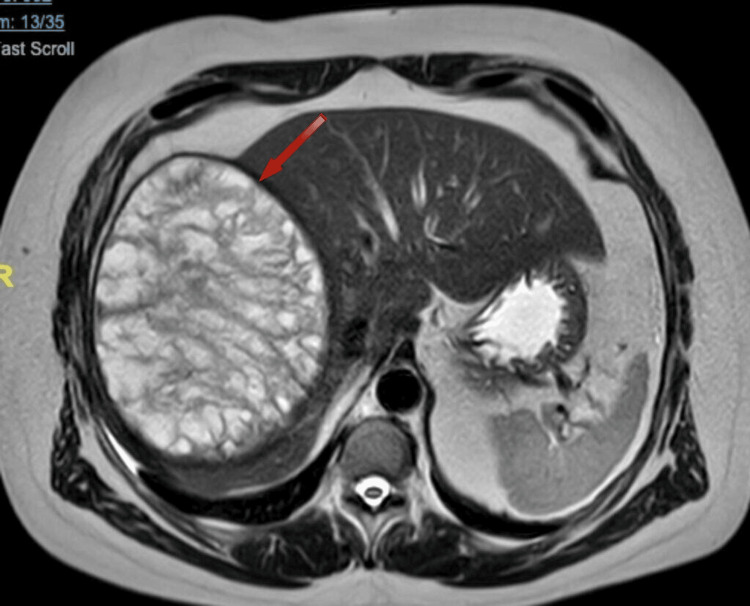
MRI axial plane Numerous irregular septa are clearly visible inside.

**Figure 4:**
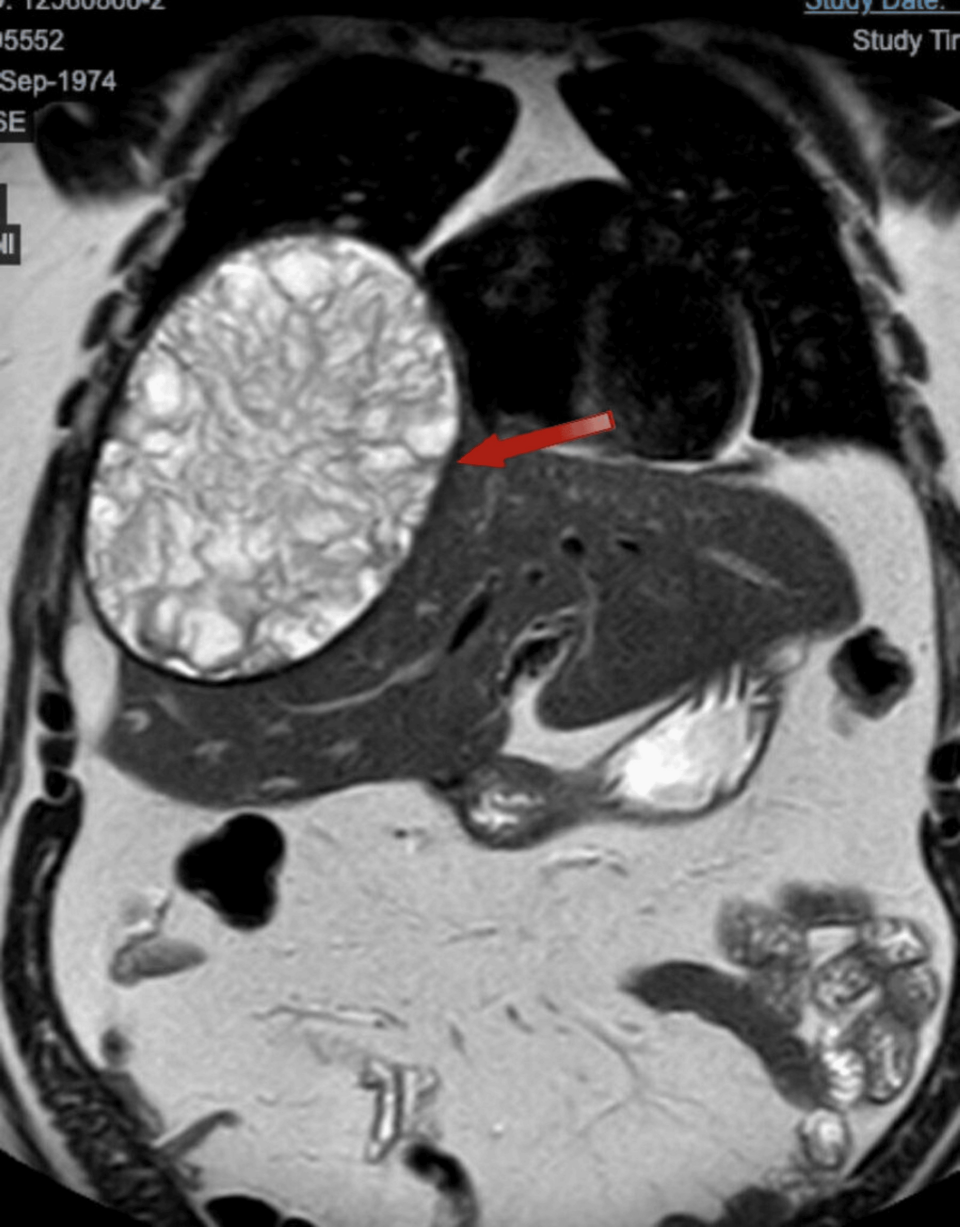
MRI coronal plane.

The magnetic resonance imaging (Figure 3, Figure 4) revealed a large cystic lesion centered in segment VIII, measuring 17 x 13 x 16.5 cm. It has a thick hypointense wall and homogeneous hyperintense signal on T1, with numerous irregular septa inside. There is no clear enhancement on contrast sequences. It causes elevation of the right hemidiaphragm, with a reduction in ipsilateral lung volume, consistent with a hydatid cyst.

Immunoglobulin G (IgG) testing for *Echinococcus granulosus* was positive. A laparoscopic pericystectomy was subsequently planned.

Video 1 demonstrates a radical approach to treating a giant hepatic hydatid cyst (>10 cm) in the right hepatic lobe through a subtotal pericystectomy. The video details the careful management of the cyst to prevent spillage into the peritoneal cavity, removal of cyst contents, and near-total resection of the pericyst to minimize residual cavities. To prevent significant hemorrhagic complications, portions of the pericystic membrane adhered to major vessels were left in situ. The visualization also includes the identification and management of biliary communications.

**Video 1:** Radical laparoscopic pericystectomy of a giant hepatic hydatid cyst

After surgery, treatment with albendazole 400 mg every 12 hours was started for three months, in cycles of 30 days. After each cycle, the patient underwent hematological and liver function tests.

The patient had no postoperative complications. He was discharged on postoperative day 3. Follow-ups were conducted at one week, two weeks, and then monthly until the albendazole treatment was completed. He progressed favorably.

## Discussion

The case presented highlights a successful laparoscopic pericystectomy for a giant hepatic hydatid cyst, emphasizing the minimally invasive approach to treating a condition that has traditionally required open surgery. Hydatid liver disease, caused by the *Echinococcus *species, is a zoonotic infection that primarily affects the liver, accounting for 50-70% of cases, often involving the right hepatic lobe due to portal blood flow dynamics. While hydatidosis is prevalent in regions with livestock farming, such as the Araucanía Region in Chile, the disease often remains asymptomatic for years, leading to incidental diagnoses upon imaging for unrelated conditions.

The approach to treating hepatic hydatid cysts varies, with open surgery historically being the mainstay. However, recent guidelines from the World Health Organization (WHO) have introduced the PAIR technique (puncture, aspiration, injection, reaspiration) for uncomplicated cases. Nevertheless, surgical management remains the treatment of choice for complex cysts or those posing significant risks, with a growing trend toward minimally invasive techniques like laparoscopy.

Laparoscopic management of hepatic hydatidosis has gained popularity in the last decade due to the numerous benefits it offers [13]. These include smaller incisions, decreased postoperative pain, shorter hospital stays, faster recovery, and improved visualization during surgery. Moreover, laparoscopy allows for precise control of vasculobiliary structures, which is crucial in cases where large cysts or difficult anatomical locations are involved [14]. As demonstrated in a retrospective analysis of 247 patients, published in 2023, laparoscopic surgery continues to play a significant role in the treatment of liver hydatid disease, with increasing utilization over the years. The findings highlight its benefits in terms of postoperative recovery, including a reduced rate of intraoperative complications [15]. However, there are still unresolved debates regarding patient selection criteria, standardization of surgical techniques, and follow-up protocols for laparoscopic procedures. Furthermore, performing laparoscopic radical procedures like subtotal pericystectomy demands advanced skills and experience in minimally invasive hepatic surgery.

## Conclusions

In this case, the patient presented with right hypochondriac abdominal pain and hepatomegaly due to a giant right-lobe hepatic hydatid cyst, which was confirmed by CT imaging and serology for *Echinococcus granulosus*. The decision to proceed with a laparoscopic subtotal pericystectomy allowed for effective removal of the cyst while preventing spillage into the peritoneal cavity and minimizing residual cavities. The case also demonstrates the careful intraoperative management of biliary communications and the conservative handling of pericystic membranes adherent to major vessels to avoid hemorrhagic complications.

The laparoscopic approach, while associated with technical challenges, showed comparable outcomes to traditional open surgery in terms of cyst clearance and postoperative recovery. The patient in this case was discharged on the third postoperative day without complications, which aligns with the advantages of minimally invasive techniques over open surgery.

The discussion of this case adds to the growing body of literature supporting laparoscopy as an effective and safe approach for the treatment of hepatic hydatidosis. It also underscores the importance of training in advanced laparoscopic skills, as this approach requires expertise in managing hepatic and cystic pathologies through minimally invasive means. While laparoscopy appears to offer significant benefits, there is a need for further studies to establish consensus guidelines on patient selection, standardized surgical techniques, and long-term follow-up protocols to optimize outcomes for patients with hepatic hydatid disease.

Overall, this case supports the evolving role of laparoscopy in the radical treatment of hepatic hydatid cysts and highlights the potential for this technique to become the preferred approach in carefully selected patients, offering comparable efficacy to open surgery with the added benefits of minimally invasive management.
